# Genome-wide identification and expression characterization of the *DoG* gene family of moso bamboo (*Phyllostachys edulis*)

**DOI:** 10.1186/s12864-022-08551-3

**Published:** 2022-05-10

**Authors:** Zhang Zhijun, Yu Peiyao, Huang Bing, Ma Ruifang, Kunnummal Kurungara Vinod, Muthusamy Ramakrishnan

**Affiliations:** 1 State Key Laboratory of Subtropical Forest Cultivation, Zhejiang A&F University, Lin’an, Hangzhou, 311300 Zhejiang China; 2 School of Forestry and Biotechnology, Zhejiang A&F University, Lin’an, Hangzhou, 311300 Zhejiang China; 3 Division of Genetics, ICAR - Indian Agricultural Research Institute, New Delhi, 110012, India; 4 Co-Innovation Center for Sustainable Forestry in Southern China, Nanjing Forestry University, Nanjing, 210037 Jiangsu China; 5 Bamboo Research Institute, Nanjing Forestry University, Nanjing, 210037 Jiangsu China

**Keywords:** *Phyllostachys edulis*, *DoG* gene family, Bioinformatics, Gene expression, BZIP transcription factor

## Abstract

**Background:**

The *DoG* (*Delay of Germination1*) family plays a key regulatory role in seed dormancy and germination. However, to date, there is no complete genomic overview of the *DoG* gene family of any economically valuable crop, including moso bamboo (*Phyllostachys edulis*), and no studies have been conducted to characterize its expression profile. To identify the *DoG* gene members of moso bamboo (*PeDoG*) and to investigate their family structural features and tissue expression profile characteristics, a study was conducted. Based on the whole genome and differential transcriptome data, in this investigation, we have scrutinized the physicochemical properties, gene structure, *cis*-acting elements, phylogenetic relationships, conserved structural (CS) domains, CS motifs and expression patterns of the *PeDoG1* family of moso bamboo.

**Results:**

*The DoG* family genes of moso bamboo were found distributed across 16 chromosomal scaffolds with 24 members. All members were found to carry *DoG1* structural domains, while 23 members additionally possessed basic leucine zipper (bZIP) structural domains. We could divide the *PeDoG* genes into three subfamilies based on phylogenetic relationships. Covariance analysis revealed that tandem duplication was the main driver of amplification of the *PeDoG* genes. The upstream promoter of these genes containing several *cis*-acting elements indicates a plausible role in abiotic stress and hormone induction. Gene expression pattern according to transcriptome data revealed participation of the *PeDoG* genes in tissue and organ development. Analysis using Short Time-series Expression Miner (STEM) tool revealed that the *PeDoG* gene family is also associated with rapid early shoot growth. Gene ontology (GO) and KEGG analyses showed a dual role of the *PeDoG* genes. We found that *PeDoG*s has a possible role as *bZIP* transcription factors by regulating *Polar like1* (*PL1*) gene expression, and thereby playing a disease response role in moso bamboo. Quantitative gene expression of the *PeDoG* genes revealed that they were abundantly expressed in roots and leaves, and could be induced in response to gibberellin (GA).

**Conclusion:**

In this study, we found that the *PeDoG* genes are involved in a wide range of activities such as growth and development, stress response and transcription. This forms the first report of *PeDoG* genes and their potential roles in moso bamboo.

**Supplementary Information:**

The online version contains supplementary material available at 10.1186/s12864-022-08551-3.

## Background

The moso bamboo (*Phyllostachys edulis)*, is a member of the genus *Phyllostachys* in the Gramineae subfamily, well known for its use as an industrial raw material. Because of its various uses in the wood, fiber, biofuel, paper, food and pharmaceutical industries, it is known as one of the most versatile herbaceous plants [1]. The flowering cycle of the moso bamboo takes around 60 years [2], and its growth phase is dominated by vegetative growth [3]. Shoot emergence is a remarkable stage in the vegetative growth of moso bamboo, which showcases the rapid growth wherein the shoots reach more than 6–7 m in 2–3 months [4]. Besides, the flowering period of bamboo is likely to be more connected to environmental stress than photo-cycling or other traditional pathways [3]. It has been shown that this period is associated with the expression of many transcription factors, such as MYB [5], bZIP [6] and NAC [7].

Seed dormancy is a genetically programmed decision to germinate seeds at the appropriate time when the environmental conditions are favorable [8]. In addition to hormones, enzymes and environmental factors [9, 10], dormancy is also controlled by several genes. Among these, a small family of genes namely *Delay of Germination* (*DoG*) is particularly noteworthy. Specifically, expressed genes such as *AtSRT2* in *Arabidopsis* involved in seed germination under salt stress [11], dormancy gene *TaSdr* in wheat [12], and *AtDoG1,* the master controller of seed dormancy in *Arabidopsis* [13, 14] signifies the genetic control of dormancy break in plants. The *AtDoG1* gene was first identified through quantitative trait locus (QTL) mapping in *Arabidopsis* [15], which was subsequently cloned [16]. *DoG1* gene is now known to work in accordance with the abscisic acid (ABA) pathway to regulate dormancy [17, 18], and influence the metabolism of hormones such as gibberellin, which acts as a negative regulator [10]. Since *DoG1* expression is sensitive to temperature, dormancy time tends to vary under changing temperature conditions [17], thereby preventing germination when environmental conditions are uncomfortable [19]. Control of germination time by *DoG* gene has also been reported in wheat [20]. Further evidence from crucifers indicates multiple roles of *DoG1 genes* such as control of early flowering [21], drought tolerance [22] etc. besides regulation of seed germination time through a temperature-sensing mechanism. However, an additional microRNAs pathway was involved in the control of early flowering [21]. Experimental evidence indicates that exogenous application of ABA and gibberellin (GA) affects *DoG* expression to varying degrees [23, 24]. A pyrimidine box (P-box) with 5’-CCTTTT-3’ is a *cis*-acting element observed in GA-responsive promoters of cereals. This motif has an important role in plants in response to GA hormone regulation of plant growth and development [25]. Additionally, the possibility of unknown functions of *DoG* in different plant growth processes could not be ruled out.

Plant transcription factors (TFs) are proteins with DNA-binding functions that exert regulatory action in gene expression by switching them on and off. TFs play a major role in transient gene expression during plant growth and development as well as in response to stresses [26]. Among the many families of TFs, the basic leucine zipper (bZIP) has a typical structural domain consisting of a basic region that contacts DNA and an adjacent 'leucine zip' that facilitates protein dimerization. The bZIP TFs are commonly active in response to plant hormones or environmental stresses [27], such as ABA [28], GA [29], salicylic acid (SA) [30], low temperature stress [31], zinc deficiency [32], drought, and antioxidants [33]. The bZIP TFs also show influence on seed germination as well as flowering [34]. Extensively studied in rice [35], *Arabidopsis* [32], maize [36] and watermelon [37], the bZIP TFs are considered to be an unequivocal regulators of plant development [27].

Since the sequencing of the moso bamboo genome in 2013 [38], there are several improvements including a reference genome [39] and several explorations of genes families. However, being an important gene family involved in seed germination to growth and development, information of the *DoG* family in moso bamboo is not available so far. In this study, we are addressing this issue through multilevel bioinformatic and wet lab studies to unfold the details of *DoG* genes in moso bamboo. We have investigated the gene structure, physicochemical properties, molecular evolution, promoter elements and transcriptome expression patterns in combined with qPCR experiments. We report here the unique features of moso bamboo the *DoG* genes in this article.

## Results

### Structural domain of DoG genes

The plant DOG protein PFAM (PF14144.3) model for 39 family members was searched, and 34 members were obtained by initial screening. Combining gene structure, protein structural domain features, and further removal of identical transcript repeats, 24 DOG proteins to be obtained that have complete conserved domains. For convenience, "*Pe*" in the representative *Phyllostachys edulis* was placed before the gene family name (*DoG*), which was named *PeDoG1* ~ *PeDoG24 in* sequence according to the order of the gene's position on the chromosome. Since we found that the *DoG* family of moso bamboo contains a structural domain of bZIP in addition to the structural domain of *DoG1*. This new finding has not been reported in other literature. However, there is reason to believe that the presence of the bZIP structural domain could confer new functional properties to the *DoG* family.

### Physicochemical properties of gene family members

The number of genes counting exons was selected in the coding region sequence file, and the amino acid sequences and physicochemical properties of these 24 family members (Table S1). The amino acid sequences and physicochemical properties of these 24 family members (Table 1) indicated that the genes encoded proteins with amino acid numbers ranging from 270 to 520, with gene numbers *PeDoG13* and *PeDoG16* having the maximum number of amino acids and gene number *PeDoG14* having the minimum number of amino acids. The molecular weights of the DOG proteins ranged from 29,387.13 to 57,397.48 daltons, with theoretical isoelectric points ranging from 5.42 to 9.27. Six genes encoded proteins with a theoretical isoelectric point less than or equal to 6.5, which is acidic, and ten genes encoded proteins greater than 8, which is basic. The aliphatic index of the DOG proteins ranged from 71.92 to 84.9, and the hydrophilic coefficients were all less than zero. All of the proteins were hydrophilic and structurally unstable (mean value of instability coefficient was 55.48). In addition, the predicted subcellular localization demonstrated that all 24 PeDOGs were localized inside the nucleus.

**Table 1:** Family member information of 24 genes of *DoG* family in *P. edulis* (Moso bamboo)

Accession number	Gene number	Number of amino acids	Molecular weight (daltons)	Theoretical pI	Aliphatic index	Grand average of hydropathicity	Instability index	Subcellular localization
PH02Gene33889.t2	*PeDoG01*	482	52,850.09	6.21	71.99	-0.51	57.33	Nucleus
PH02Gene17660.t1	*PeDoG02*	477	53,052.89	6.84	76.58	-0.525	56.44	Nucleus
PH02Gene43974.t1	*PeDoG03*	296	32,851.41	9.26	80.51	-0.483	58.95	Nucleus
PH02Gene05301.t1	*PeDoG05*	296	47,264.43	7.86	80.51	-0.483	59.46	Nucleus
PH02Gene38060.t1	*PeDoG04*	434	37,001.63	8.56	83.53	-0.284	54.35	Nucleus
PH02Gene44830.t1	*PeDoG06*	330	56,167.42	5.99	77.58	-0.643	54.54	Nucleus
PH02Gene28154.t1	*PeDoG07*	363	40,133.5	8.99	83.69	-0.332	53.56	Nucleus
PH02Gene44454.t2	*PeDoG08*	442	48,593.22	9.06	76.52	-0.486	60.4	Nucleus
PH02Gene37785.t1	*PeDoG09*	485	53,367.89	8.32	74.35	-0.509	71.04	Nucleus
PH02Gene39181.t1	*PeDoG10*	485	53,275.63	6.71	71.92	-0.52	48.27	Nucleus
PH02Gene26016.t1	*PeDoG11*	483	53,264.83	6.71	74.22	-0.488	57.56	Nucleus
PH02Gene33148.t1	*PeDoG13*	520	57,397.48	6.47	72.6	-0.481	54.68	Nucleus
PH02Gene37919.t1	*PeDoG12*	471	51,609.06	6.86	80.96	-0.439	55.22	Nucleus
PH02Gene39868.t1	*PeDoG14*	270	29,387.13	5.42	75.00	-0.398	54.88	Nucleus
PH02Gene07819.t1	*PeDoG15*	334	37,197.08	8.9	83.11	-0.532	56.11	Nucleus
PH02Gene04519.t4	*PeDoG17*	486	53,603.15	8.33	77.22	-0.529	54.25	Nucleus
PH02Gene15488.t1	*PeDoG16*	520	57,198.28	6.5	74.13	-0.462	51.7	Nucleus
PH02Gene16035.t1	*PeDoG18*	330	36,974.61	7.74	79.94	-0.614	47.57	Nucleus
PH02Gene31633.t1	*PeDoG19*	382	41,826.25	6.57	77.85	-0.358	57.06	Nucleus
PH02Gene28667.t1	*PeDoG20*	334	37,340.2	9.27	82.81	-0.562	54.66	Nucleus
PH02Gene24017.t3	*PeDoG21*	404	45,269.62	8.49	80.27	-0.455	55.29	Nucleus
PH02Gene25703.t1	*PeDoG22*	334	37,255.11	8.92	83.38	-0.526	56.94	Nucleus
PH02Gene47106.t1	*PeDoG23*	403	44,529.46	6.78	77.67	-0.387	53.17	Nucleus
PH02Gene36596.t1	*PeDoG24*	392	44,147.16	6.15	84.9	-0.426	48.13	Nucleus

### Chromosome-wise distribution and inter-genomic and covariate relationships

**Fig. 1:**
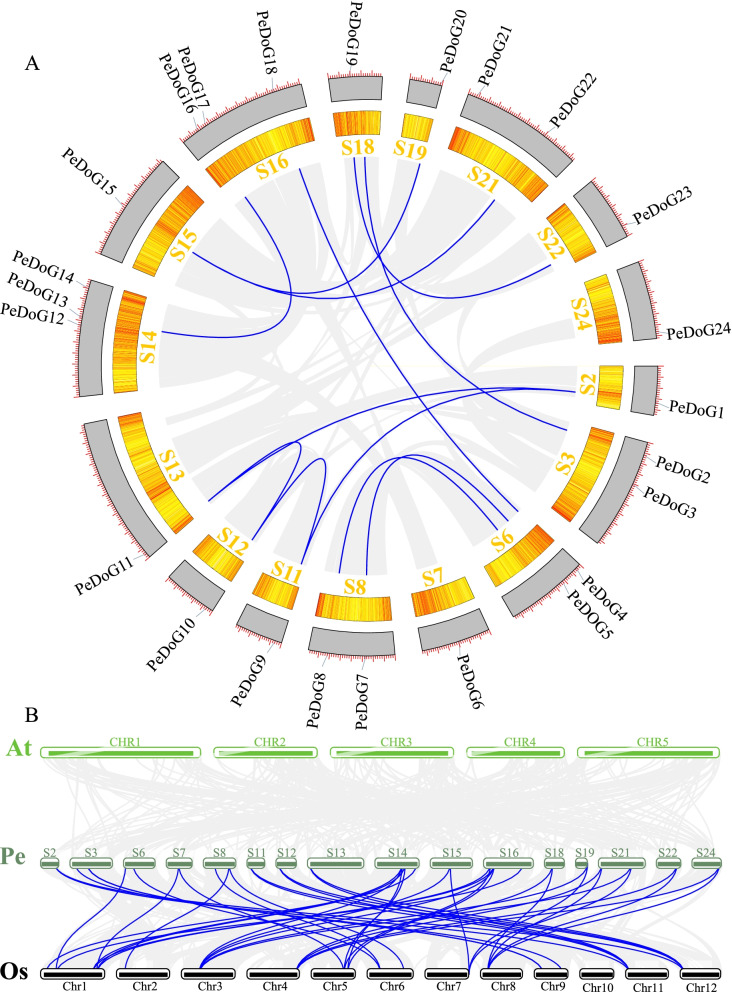
Analysis of chromosome distribution and intra-syntenic relationship of *DoG* family in *P. edulis* (Moso bamboo)*.* **A**: The chromosomal distribution of the *DoG* gene in *P. edulis.* Gray lines indicate collinear relationship of all the members of *P. edulis*, blue lines represent collinear relationship between the members of *DoG* family and yellow areas show the gene density; S: chromosome Scaffold of moso bamboo*.* **B**: Interlinear analysis of *P. edulis* (Pe), *Arabidopsis thaliana* (At) and *Oryza sativa* (Os). CHR, Chr: chromosome

Homologous genes generally have similar gene structures and biological functions [35], and therefore gene duplication plays an integral role in determining gene functions. The 24 *PeDoG* genes were found distributed across 16 linkage groups of the moso bamboo genome. Eight linkage groups (S1, S4, S5, S9, S10, S17, S20 and S23) did not include any *DoG* sequences. The Circos plot revealed that the genes were unevenly distributed on the chromosome backbone (Fig. 1A). The linkage groups S14 and S16 were the most distributed, with three *DoG* genes each. Linkage groups S3, S6, S8 and S21 contained two genes each, and the remaining ten linkage groups contained one *DoG* gene each. A total of 10 pairs of tandem repeats with good co-linearity occurred among the *PeDoG* family, with linkage group S6 having three pairs of tandem gene clusters (Fig. 1A). A two-by-two comparison of the genes encoding the DOG proteins revealed that six genes, including all genes in the two chromosome backbones, were not covariant. The covariance of the *PeDoG* genes with rice and *Arabidopsis* revealed that all the *DoG* gene members could be found with corresponding paralogs on 12 chromosomes in rice, but no covariate was found in *Arabidopsis* (Fig. 1B). The *DoG1* gene was more homologous to rice than to *Arabidopsis*. Twenty-two *PeDoG* genes had two homologous copies on the rice chromosome, and only two genes had one copy.

### Phylogeny tree of *DoG* genes

**Fig. 2:**
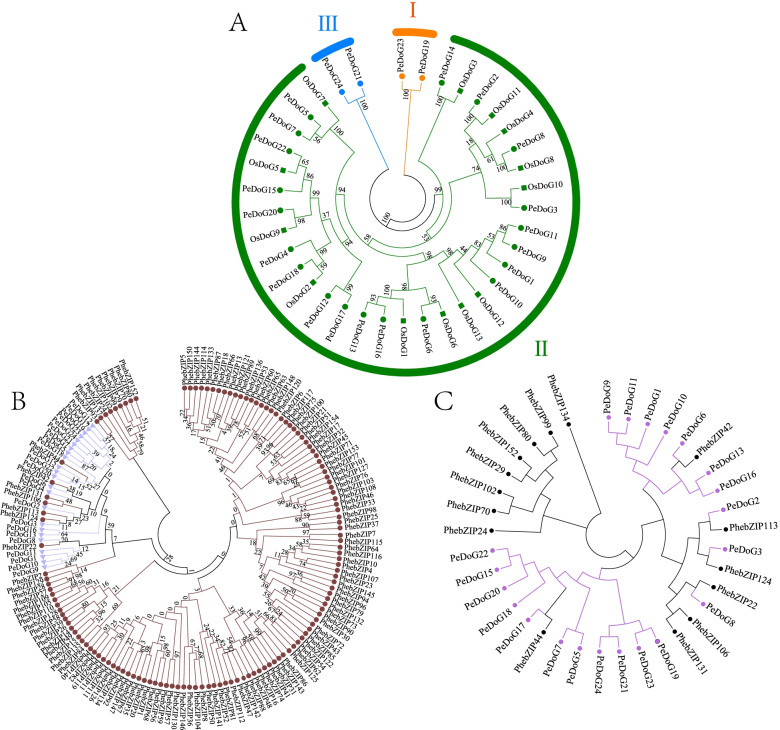
Phylogenetic analysis of *DoG* gene family from *P. edulis* (Pe) (Moso bamboo) and *O. sativa* (Os). **A**: Solid circles represent DOG family proteins of *P. edulis (Pe)*; solid rectangles represent *Oryza sativa*’s (Os) DOG family proteins. **B**: solid circles denote bZIP family proteins, solid triangles denote DOG family proteins, where some renamed bZIP families are named with DOG. **C**: Evolutionary tree of bZIP family subfamily C. Solid black circles indicate bZIP family proteins and solid purple circles indicate DOG family proteins, where some renamed bZIP families are named after DOG

To understand further the evolutionary classification of the *PeDoG* family, sequences of 13 known rice DOG proteins were compared. Based on the topology of the evolutionary tree (Fig. 2A), three subclades, I, II and III were identified. Both subclade I and subclade III had two family members, while subclade II had 20 members. Subclalde II proteins were closely related to the rice *DoG* family (Table S2), and subclade I and subclade II proteins remained distinct. Further comparison with bZIP proteins identified 23 *PeDoG* genes showing similarity to the known sequences of the *PhebZIPs* such as *PhebZIP22*, *PhebZIP113*, *PhebZIP124*, *PhebZIP106*, *PhebZIP131*, *PhebZIP42*, and *PhebZIP44*. The dendrogram shows that all except *PeDoG14* of the *PeDoG*s belong to the C subclade of bZIPs (Fig. 2B, and C).

### Family promoter characteristics

**Fig. 3:**
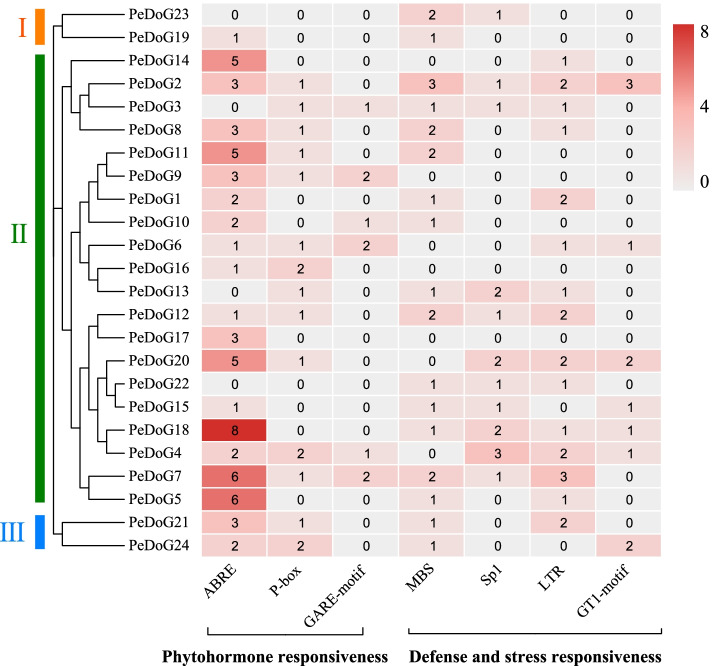
Analysis of *Cis*-acting elements on promoters of *DoG gene* family in *P. edulis* (Moso bamboo). The color scale on the right side indicates the number of *Cis-acting* elements per gene

To better understand the regulatory functions of the promoters of the *PeDoG*s the transcriptional level and the functional differences between the promoters of different members, the 2000 bp upstream promoter sequences were extracted. Analysis for three hormone-related *cis*-acting elements and four stress-responsive elements (Fig. 3), indicated that the upstream regions of all *PeDoG* genes were found to contain at least one phytohormone response. Common phytohormone responses tested were abscisic acid response (ABRE), gibberellin response Pyrimidine-box (P-box), and GA responsive element (GARE)-motif. ABRE was the most widely distributed element, with the subclade II having the maximum distribution of ABRE elements with 2.85 per gene. All the three subclades had ABRE elements distributed within, while P-box elements could be seen only in subclades II and III, while GARE-motif was confined only to subclade II. There were eight ABRE elements in *PeDoG18*. Two genes, *PeDoG7* and *PeDoG5* had six ABRE elements each, while *PeDoG14*, *PeDoG11* and *PeDoG20* contained five elements each. Other phytohormone response elements, P-box and GARE-motifs were relatively low. In addition, all family members are shown to contain one or more defensive and stress-responsive elements, such as MYB binding site (MBS)- a drought response element, a low temperature-responsive (LTR) element and light response element (Sp1).

### Analysis of conserved structural domains and conserved motifs

**Fig. 4:**
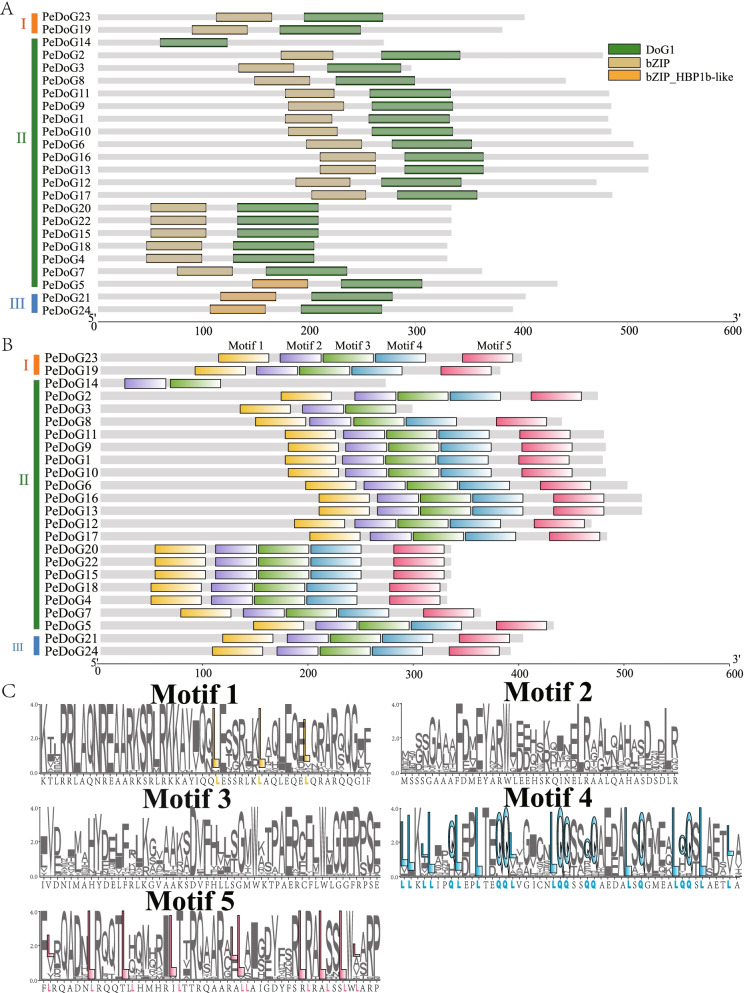
Analysis of conserved structural domains and conserved motifs of *PeDoG*s in *P. edulis* (Moso bamboo). **A**: Domains of *PeDoG*s predicted by NCBI-CD D. **B**: Motifs of *PeDoG*s predicted by MEME*.* **C**: The yellow amino acids in Motif indicate the amino acid sequences that match the structural features of bZIP, while the red and blue amino acids indicate the motif3 and motif4 feature sequences, respectively

Analysis of the conserved structural domains and exon–intron organization of *PeDoG*s (Fig. 4A), revealed that all family members contained the *DoG* domain. Two additional domains were also identified among 23 genes, the *bZIP* family and the *bZIP*_HBP1b-like structural domain. One gene, *PeDoG14* did not have any additional domains. The *bZIP* domains were found among 20 genes (83%) of the genes while three genes (12.5%) contained the bZIP_HBP1b-like structural domain. All members of Subclade I carried bZIP superfamily structural domains, whereas all the members of subclade III contained bZIP_HBP1b-like domain. The subclade II genes were carriers of either of the domains, with 90% of the genes having the bZIP domain.

There were five different motifs found on the *PeDoG*s (Fig. 4B). Although most of the genes contained all the five motifs*,* there were genes with a lesser number of motifs. Sorting the motifs according to their number of occurrences in the *DoG* families showed that Motif 2 and Motif 3 (E-values are respectively 6.9E^−773^ and 1.3E^−423^) were present in 24 genes, while Motif 1 and Motif 4 (E-values are respectively 1.7e^−694^ and 4.0e^−583^) were present in 22 genes and Motif 5 (E-value is 7.4e^−761^) was present in 23 genes. It is evident that all the family members are homologous and share conserved motifs. Combining the information from the conserved structural domains (Fig. 4A), it becomes clear that Motif 1 and Motif 5 are *DoG1* structural domains, whereas Motif 1 contains the bZIP structural domain (Fig. 4C). The structural point of the bZIP family is the leucine zip region involved in oligomerization closely linked to the basic region [40]. While a large amount of leucine was found in Motif 5, a large amount of alanine and leucine was present in motif 4.

### Three-dimensional protein sequence homologous modelling

**Fig. 5:**
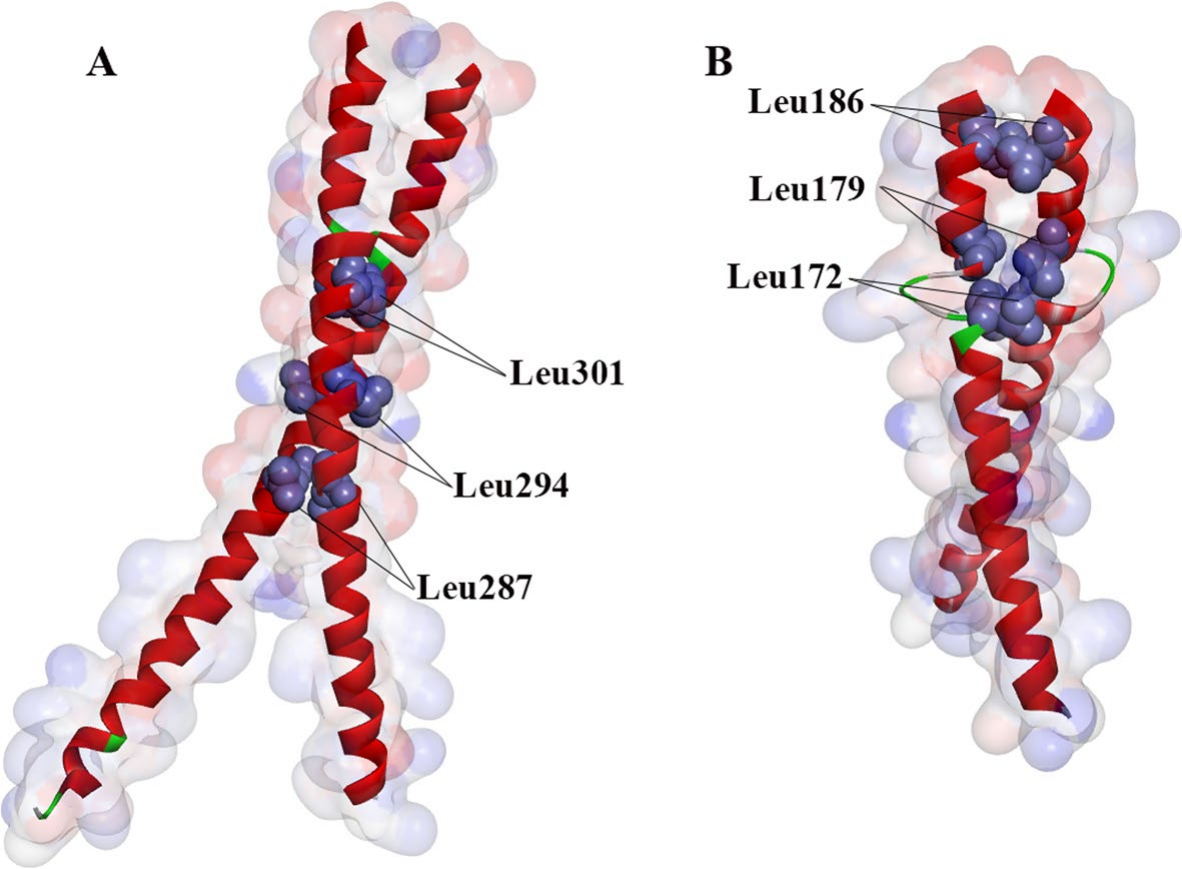
3D homology modeling of protein sequences analysis of *DoG* gene family in *P. edulis* (Moso bamboo)*.* **A**: *PeDoG1*. **B**: *PeDoG3*; The purple orbs in A and B indicate the leucine positions

*PeDoG1* (homologous to *PhebZIP3*) and *PeDoG3* were selected for modelling and visualization. The protein tertiary structure homology modelling showed (Fig. 5) that the protein sequences of the *DoG* family have the characteristic structural domains of bZIP: Lys 287, 294, 301 in *PeDoG1* and Lys 172, 179, 186 in *PeDoG3*, which are consistent with the structural feature of one leucine for every six amino acid intervals for a total of three leucine (L-X6-L-X6-L) and are close to the N-terminal, which is in accordance with the conserved motif result.

### Prediction and enrichment analysis of the *PeDoG* genes

**Fig. 6:**
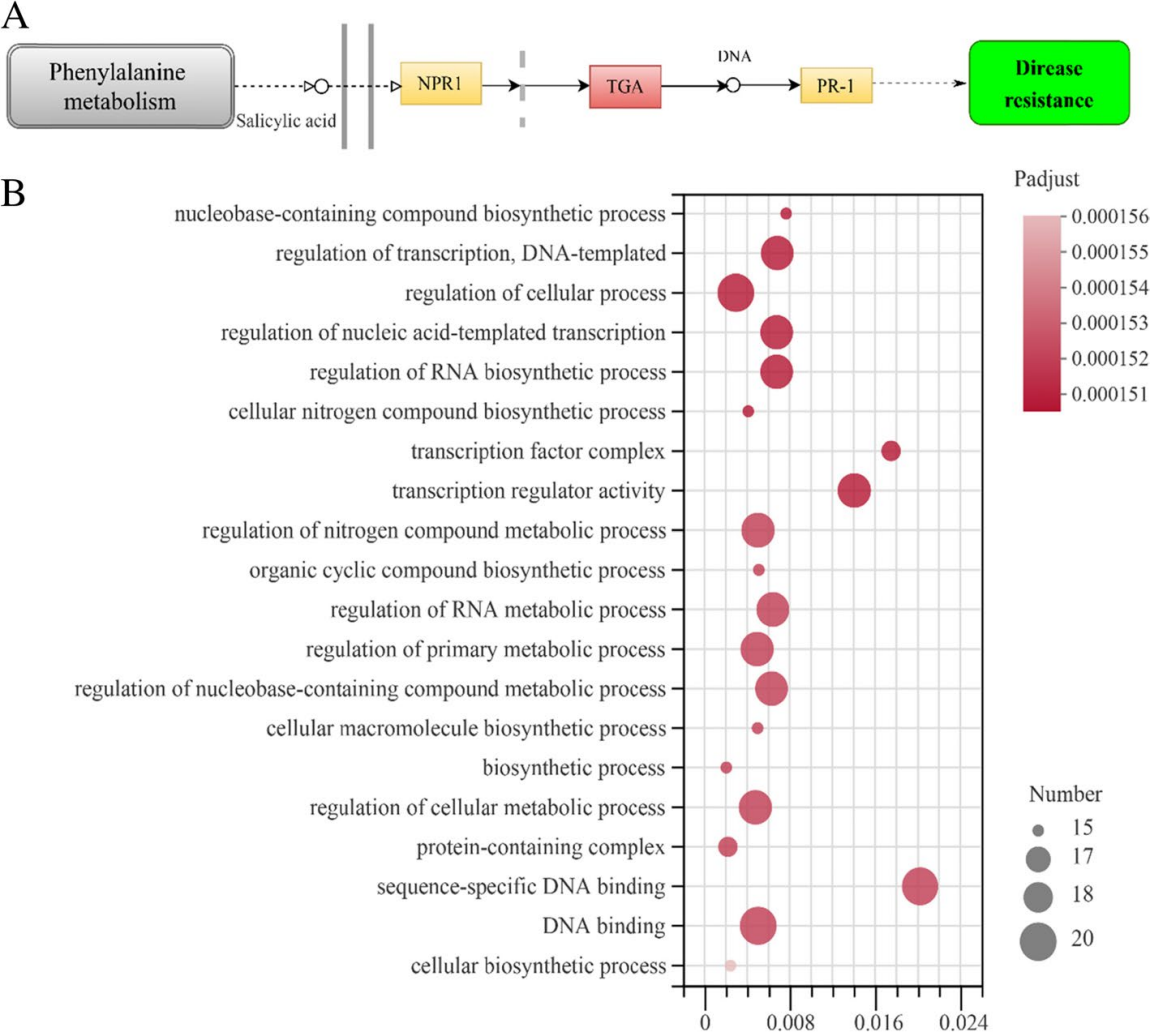
Differential expression of genes involved in hormone signaling pathways and bubble diagram of gene ontology (GO) terms. **A**: Differential expression of *PeDoG* genes in vitro, colors indicate the expression values of the genes. Expression values are presented as TPM values lg10 transformed counts, red boxes indicate genes in which family members are involved, yellow indicates monomers in which family genes are not involved. **B**: GO enrichment analysis of *PeDoG* genes in the top 20; vertical axis indicates GO terms; horizontal axis indicates Rich factor. the larger the Rich factor, the stronger the enrichment. The size of the dots indicates the number of genes in the GO terms

The conserved domains analysis suggests that *PeDoG* family could also be presumed as a subfamily of *PebZIP* family. From the PlantTFDB 4.0 TF prediction, it is identified that all the twenty-three TFs are TGACG-Binding (TGA). The Kyoto encyclopedia of gene and genomes (KEGG) analysis showed that these are involved in plant hormone signal transduction (Table S3). The classification of 23 genes into functional groups using gene ontology (GO) showed that *PeDOG*s were allocated to three GO categories (Fig. 6): biological process, cellular composition and molecular function. Enrichment analysis of the three categories was performed to select the top 20 entries with the highest significance levels, with two categories of cellular composition entries, three categories of molecular function entries and 15 categories of biological process entries. In the cell composition category, there were protein-containing complexes (GO:0,032,991) and transcription factor complexes (GO:0,005,667), while under the molecular function category, there was transcriptional regulator activity (GO:0,140,110), specific DNA binding (GO:0,043,565) and DNA binding (GO:0,003,677).

In the biological process, the genes were found to be associated with basic biological regulation (GO:0,006,355), cellular processes, metabolic processes, and most notably in the biosynthesis of cellular nitrogen compounds (GO:0,044,271). Additional functions included regulation of nucleic acid-regulated transcription (GO:1,903,506), regulatory mechanisms of ribonucleic acid biosynthetic processes (GO:2,001,141), regulation of cellular processes (GO:0,050,794), regulation of transcription and DNA-templating (GO: 0,006,355), and biosynthetic processes of nucleobase-containing compounds (GO:0,034,654). GO enrichment analysis shows that *PeDoGs* are distributed in different proportions in several significant groups in all three GO classes (Table S4).

### Predictive analysis of protein interaction networks

**Fig. 7:**
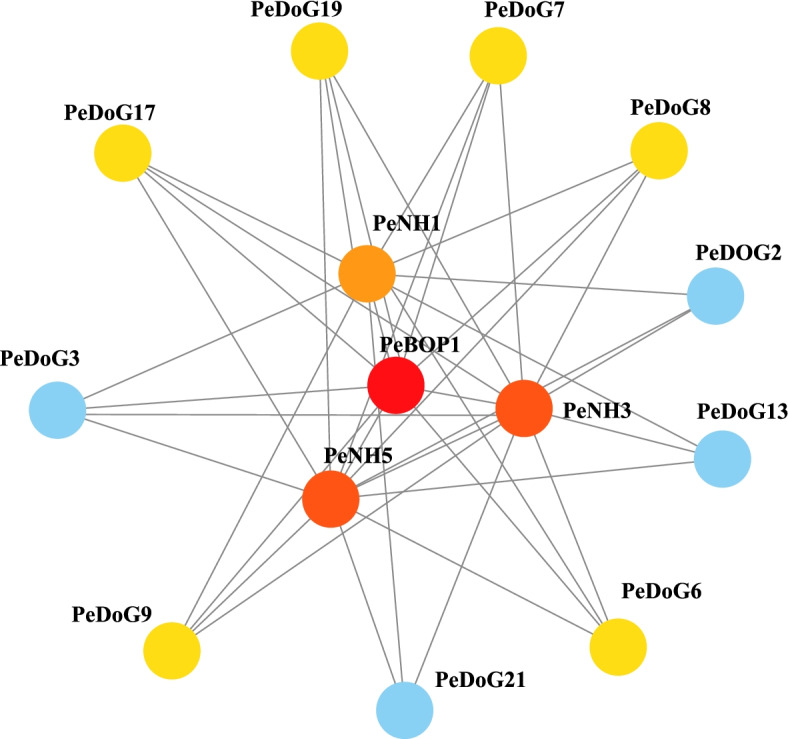
Protein–protein interaction (PPI) network of the PeDOGs. The nodes are all the core proteins of the hormone signaling pathway, and the gray connecting lines represent the predicted protein interactions, with the color gradually increasing from dark (blue) to light (red), indicating a gradual increase in the number of interacting genes

Predictive analysis on the function of the *PeDoG* family of genes, a protein–protein interaction (PPI) network was constructed based on the STRING download data. A total of 13 significant nodes and 42 interactions were found (Fig. 7). Ten members of PeDOG could interact with PeBOP1, PeNH1, PeNH3 and PeNH5 proteins (Table S5).

### Expression of *DoG* family genes (transcriptome) in different organs of moso bamboo

**Fig. 8:**
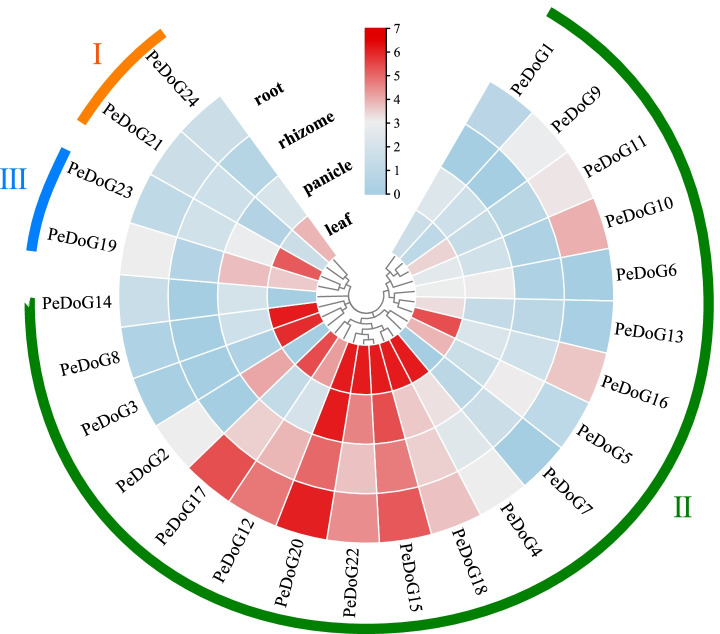
Transcriptome expression of *DoG* family in different stages and different organs of *P. edulis* (Moso bamboo). The scale value is from low to high, and the color changes from blue to red, which represents the expression from low to high

The *PeDoG* gene expression patterns in root, rhizome, panicle and leaf tissues of moso bamboo, transcriptomic data (ERR105067, ERR105069, ERR105073, and ERR105075) from the EMBL database (http://wwwdev.ebi.ac.uk/), indicated stronger gene expression in leaves followed by roots and rhizomes (Fig. 8). The heatmaps of transcripts per million (TPM) showed that each gene is expressed in at least two of the four organs, with 16 genes expressed in every tissue tested. Among the genes belonging to subclades, leaf and panicle expression were prominent for clades I and III, while clade II genes were found to express in every organ, but with different levels. Particularly, *PeDoG20*, *PeDoG15*, *PeDoG18* and *PeDoG22* show high expression in every organ, with the strongest expression in leaves. In addition, some genes were expressed in most of the tissues analyzed and some were barely expressed except in one particular organ, indicating their tissue specificity. *PeDoG24* in subclade I, *PeDoG23* in subclade III*, PeDoG4, PeDoG3, PeDoG16, PeDoG8* and *PeDoG13* in subclade II were significantly more highly expressed in leaves; *PeDoG1, PeDoG2, PeDoG7* and *PeDoG14* in subclade II exhibited high expressed in the inflorescence, which indicates that four genes are related to flower. *PeDoG9, PeDoG10 and PeDoG12* were more highly expressed in roots.

### qPCR expression levels of the *DoG* family in different tissues

**Fig. 9:**
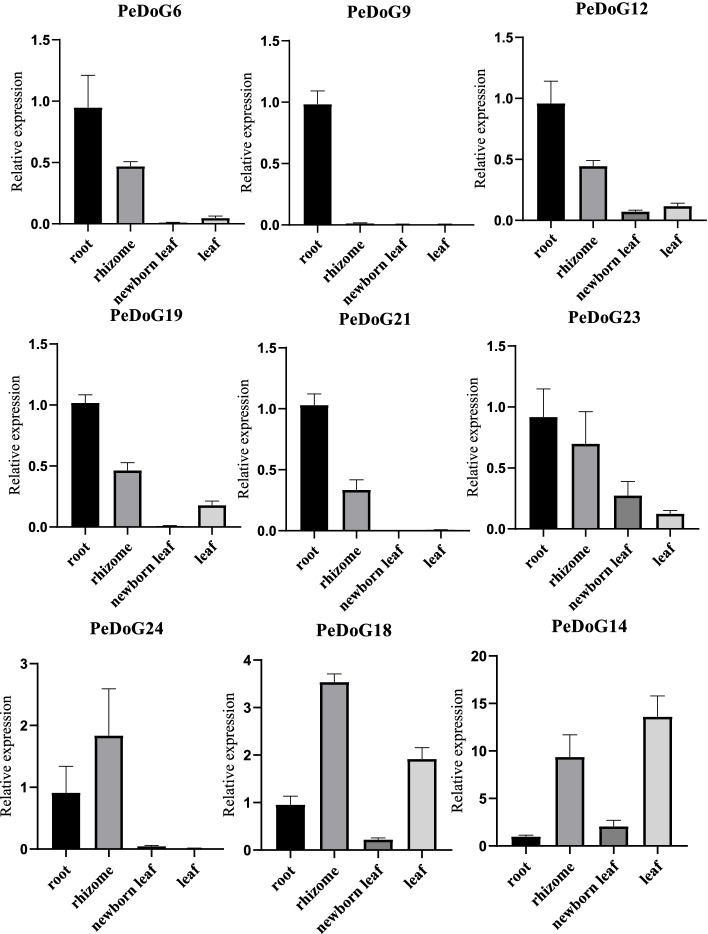
Quantitative Real-time PCR analysis from a part of *DoG* family in different organs (different tissues from field samples) of *P. edulis* (Moso bamboo)

To validate the reliability and consistency of the transcriptome data of *DoG* family, we have carried out quantitative real-time polymerase chain reaction (qRT-PCR) to further examine the expression patterns of genes belonging to three subclades (Fig. 2A), in different tissues such as root, rhizome, new flush and mature leaf. The qRT-PCR expression patterns (Fig. 9) of most of the *DOGs* selected were similar in each subclade, except for subclade II genes which showed differential expression patterns. Subclade III genes, *PeDoG21* and *PeDoG24* were found not expressed leaves, but a substantial expression could be noticed in root and rhizomes. The subclade I genes, *PeDoG19* and *PeDoG23,* were also found highly expressed in roots, followed by rhizome and leaf, but the former was not noticed expressed in the flush. Among the subclade II genes, *PeDoG14*, showed high leaf expression followed by rhizome while it was barely expressed in roots and early flush. Another gene of the same subclade, *PeDoG18* exhibited high expression in rhizome tissues followed by leaf but had low expression in roots and young flush. Interestingly, *PeDoG9*, was found only expressed in roots and no other tissues. The remaining genes, *PeDoG6*, *PeDoG12*, and *PeDoG21*, were statistically found prominently expressed roots followed by rhizome, while their leaf expression looked insignificant.

### Expression analysis of the *DoG* family genes in seedlings of moso bamboo in response to GA

**Fig. 10:**
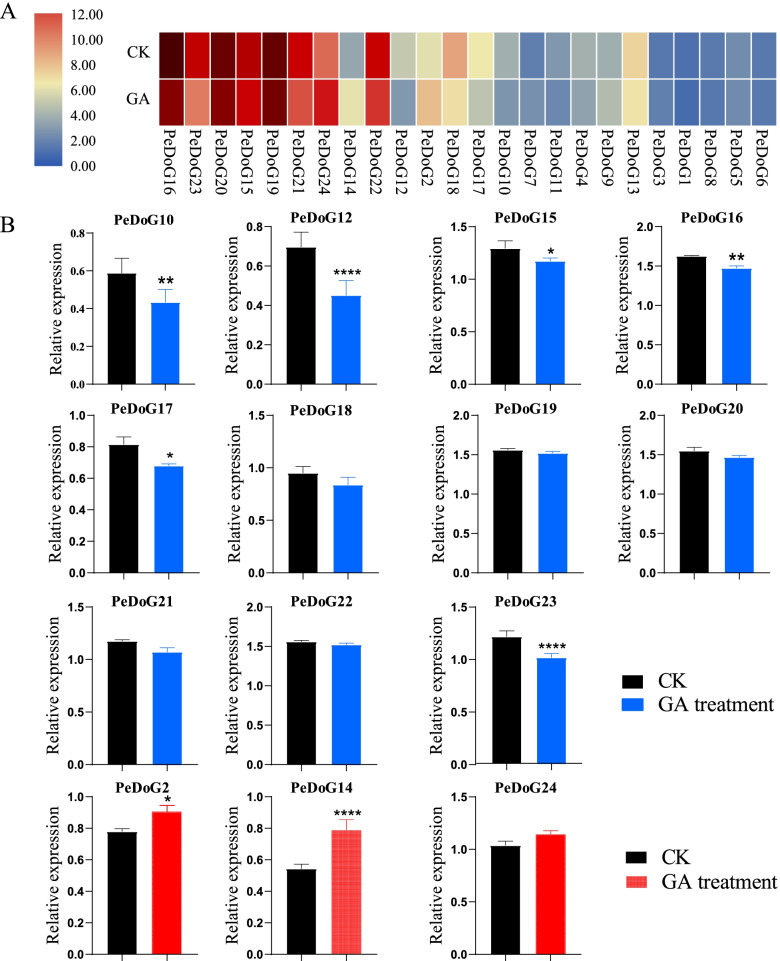
Seedling's relative expression levels in gibberellin treatment of *P. edulis* (Moso bamboo)*.* **A**: Relative expression of each gene of *PeDoG* in GA treatment; **B**: GA-treated red bars with red boxed lines indicate an increase in the expression of the gene to GA and black boxed lines blue bars indicate a decrease in the expression of the gene to GA; *, *p* < 0.05; **, *p* < 0.01; ****, *p* < 0.0001. Three biological and three technical replicates were used for each real-time PCR

To analyze whether the *PeDoG*s responded to exogenous GA, we recruited 14 genes with high variability (Fig. 10A), which were ranked by the magnitude of the differences as *PeDoG10, PeDoG17, PeDoG18, PeDoG2, PeDoG12, PeDoG22, PeDoG14, PeDoG24, PeDoG21, PeDoG19, PeDoG15, PeDoG20, PeDoG23,* and *PeDoG16*. Among the 14 selected genes, we found that the relative expression of three genes *PeDoG2, PeDoG14 and PeDoG24* was up-regulated after GA treatment. Although the increment with *PeDoG24* was insignificant, the other two genes had a significant level of expression. The remaining genes had a downregulation trend in expression, and those with significant differences were *PeDoG10, PeDoG12, PeDoG15, PeDoG16, PeDoG17* and *PeDoG23.* Among these, two genes with remarkable downregulation were *PeDoG23* and *PeDoG12* (Fig. 10B).

### Time series expression analysis

**Fig. 11:**
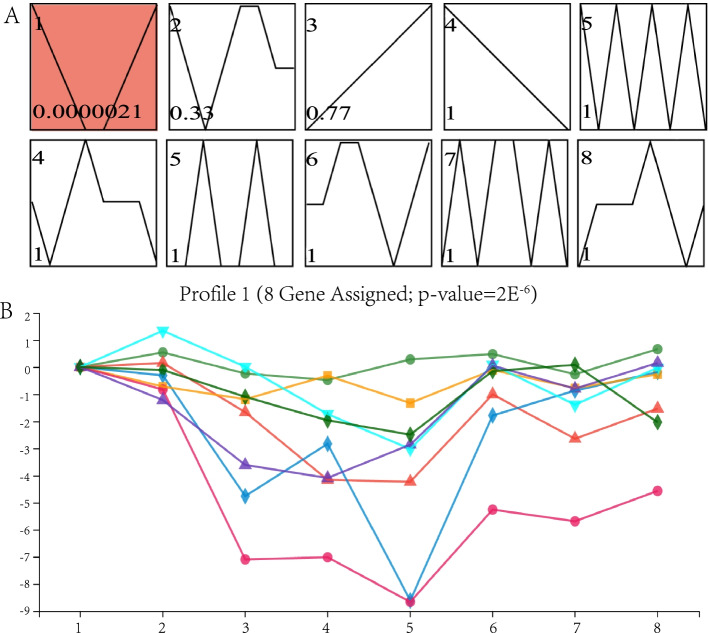
The Short Time-series Expression Miner (STEM) analysis of *PeDoG* family. A: trend graph of 10 genes with changed expression trends, red trend graph indicates that the temporal pattern of the profile conforms to the significant change trend; colorless trend graph indicates that the temporal pattern of the profile is a statistically non-significant change trend; B: trend graph of all genes under the profile with a p-value of 2.1E^−6^

The Short Time-series Expression Miner (STEM) is a tool in genetic analysis for comparing and visualizing temporal expression data. STEM report of the *PeDoG* family at different stages (Fig. 11) showed a singular gene expression pattern, indicating that expression of most of the *PeDoG*s initially declined with the shoot growth reaching the lowest point at stage 5 (5 m growth height), and then elevated significantly. With a total of eight members showing a similar trend in expression pattern, we could infer that *PeDoG*s are involved in the growth process.

### Subcellular localization of the PeDOG protein

**Fig. 12:**
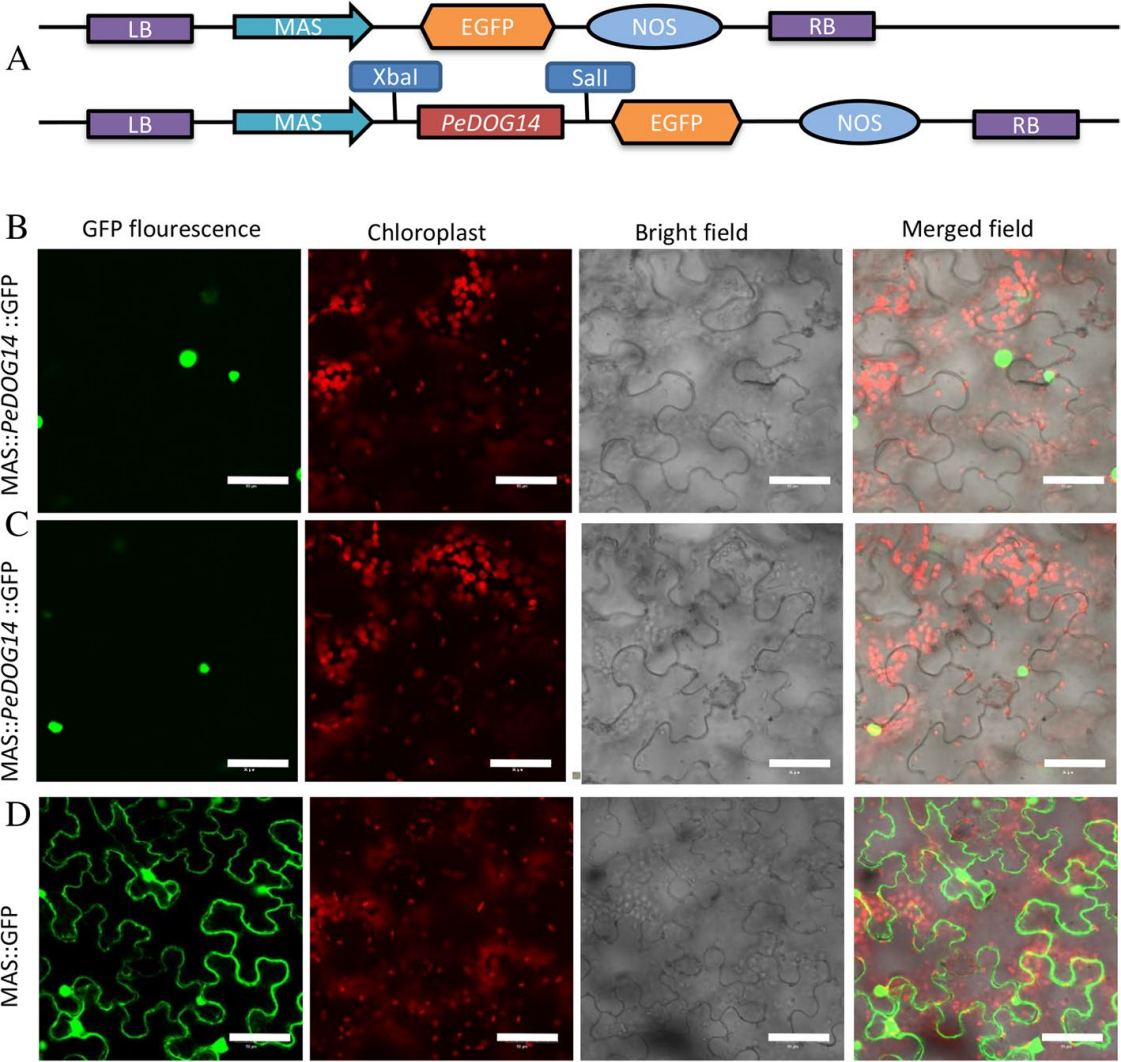
Subcellular localization of GFP-*PeDOG14* by transient expression in the cells of tobacco leaves. **A**: Schematic diagram of the DNA construct used for PeDOG14 subcellular localization. LB, T-DNA left border. MAS, cauliflower mosaic virus MAS promoter; GFP, green fluorescent protein; NOS, nopaline synthase gene terminator; RB, T-DNA right border. **B**-**C**: Subcellular localization of GFP-tagged PeDOG14. **D**: Subcellular localization of GFP control

To determine the subcellular localization of the PeDOG proteins in moso bamboo, we first created the P1300-MAS-PeDOG14-GFP construct (Fig. 12 A). After driving through the MAS promoter (cauliflower mosaic virus), the CDS full-length of *PeDOG14* was fused to GFP and expressed in tobacco epidermal cells. The GFP green fluorescent signal was used to determine the position of PeDOG14 protein in the cell. GFP null was used as a control (Fig. 10B). PeDOG14 was discovered clearly to be a nuclear-localized protein (Fig. 12B-D).

## Discussion

Our analysis supports the hypothesis of long-term regulation of *PeDoG* gene expression by abscisic acid (ABA). However, the different numbers of elements among *PeDoG* genes suggest different expression patterns in response to a variety of plant hormones and transcription factors. For example, ABRE, the corresponding ABA signaling element, includes an ACGT nucleotide core motif that can be identified by the bZIP transcription factor [41]. TGA is an important regulator of salicylate SA (salicylic acid) induction and contains the conserved sequence TGACG, which belongs to the bZIP family of transcription factors [41]. TGA interacts with NPR1 and binds to the SA response element of the PR-1 promoter [42], thereby activating downstream genes and ultimately participating in the disease resistance response.

BOP1 controls ribosome biogenesis and cell cycle progression [43], the disease resistance regulatory protein NPR1[44], the rest of the PeNH family are homologues of NPR1 and interact with TGA, NH1 and NH3 proteins are involved in the immune response of plants and all three proteins are associated with plant disease resistance [45], corresponding to the results in the pathway analysis. These genes with high expression levels may also be housekeeping genes of the plant, mainly related to leaf activity. There were more significant differences in all three genes when compared to the control group, demonstrating that the *DoG* family is very closely associated with the regulation of GA metabolism. The decreasing expression may be due to the role of the family genes in controlling seed dormancy, with the family members gradually becoming less expressed as the moso bamboo grows; the subsequent increase in expression is evidence of their involvement in moso bamboo growth activities, especially at the shoot and bud development stages.

In plants, *DOGs* are found to be a small gene family with relatively few members. Although small, *DoG*s are considered an important gene family that perceives environmental fluctuations to signal stage specific and stress specific gene expression. The role of *DoGs* has already been established in *Arabidopsis* seed germination, as well as in other species such as rice, wheat, barley and maize [32, 34–36]. Being an important regulator of germination, these genes occur in large numbers and show constitutive expression indicating their additional role in plant growth and development. However, no information is available on this important gene family in moso bamboo and their potential role. It has been already reported that *DoG* family in *Arabidopsis* has five members, while 12 genes are in rice. Surprisingly, we could notice a total of 24 *DoG*s in moso bamboo, the largest so far reported in any species, and most members have two protein structural domains, and all family members have DOG protein structural domains, indicating a relatively conservative evolutionary pattern.

Despite the fact that moso bamboo has twice the number of genes as rice, we speculated a genome wide synteny among the genes. Homologous genes sharing similar or altered functions originating from speciation (orthologs) and duplication (paralogs) are common among living organisms [46]. In many cases, the number of members of the interspecific gene family is related to the size and complexity of the interspecies genome, because of the accumulation of paralogous genes. We found that the 24 *DoG*s of moso bamboo were separated into three classes as found by three subclades. Homology analysis in comparison with rice and *Arabidopsis DoGs* identified that the *PeDoGs* were closer to *OsDoGs*, but synteny was only identified with the class II genes and not with the Class I and III genes. A significant departure from *AtDoG*s was noticed, possibly indicating the cladal divergence; *Arabidopsis* belongs to the Dicotyledonae clade, while both moso bamboo and rice are monocotyledons. Therefore, rice and moso bamboo could maintain a higher interspecific homology and stronger gene family affinity. In addition, the high number of *PeDoG*s could probably be due to the apparent genome polyploidization in moso bamboo, which resulted in the amplification of gene families. Evolutionary history indicates that bamboo genomes remain as close to that of the cultivated grasses, wheat, rice, and sorghum separated by 47, 49 and 65 million years ago, respectively with a possible tetraploidization through a duplication event that occurred 7–12 million years ago [38]. Homologous genes with similar sequences are usually similar in function. Divided into three classes, the *PeDoG*s exhibited relatively large changes in the physicochemical properties. These changes could have a result of speciation events when the moso bamboo went through evolutionary adaptation [47] for being acclimatized to temperate ecology deviating from the tropical adaptation of cereals like rice and sorghum as well many other bamboo species. However, the structural domains, motifs and spatial 3D conformations of the proteins of members of the same subfamily show a more or less conserved pattern, implying similar functionality. The fact that all the genes have DoG domains and most of the members have additional bZIP structural domains, indicates a relatively conservative evolutionary pattern of *PeDoG*s.

Interestingly, unlike *DoG* families in related grasses, the *PeDoG*s were enriched with a bZIP structural domain, indicating multiple roles they may play. The sequence homology modelling also revealed that the bZIP domains of *PeDoG* are functional because they share the same structural features of a basic leucine zipper. At the same time, analysis of conserved motifs revealed that some fragments have a large amount of leucine as well as alanine, and there may be a possibility of additional functional features. We could demonstrate structural relations with 21 of the *PeDoG*s with *PhebZIP*s. *PhebZIP*s are bZIP TFs of moso bamboo, with 154 genes falling in nine sub-families that are majorly involved in growth and development particularly in seed development [6]. Strikingly, all the *PeDoG*s were aligned closer to the subfamily C of *PhebZIP*s indicating that this subfamily of bZIPs is the same that carried *DoG* domains in moso bamboo. Further analysis by transcription factor identification and GO annotation concluded that the *PeDoG* family can act as a bZIP family transcription factor and can be involved in the SA signalling pathway, directing plant shoot and bud growth as well as in biotic stress response such as in plant disease resistance. We, therefore, conclude that besides the basic *DoG* functionality, the *PeDoG*s take an inextricably linked role of the *PhebZIP* family of genes. Expression studies with the selected *PeDoG* genes in response to abiotic stresses further corroborates the biological functions of the promoters. GO terms also indicate an association with rapid growth of bamboo [48]. Protein interactions network of *PeDoG*s was also indicative of their potential role in plant defence responses in addition to seed dormancy and can act in the plant immune response. Further, the network also threw light on a role in the growth and developmental processes of moso bamboo, emphasizing the observations made from the GO reports.

The *PeDoG* genes also had an array of variations for the *cis*-acting elements they carry at the upstream promoter sequence. The most common element was ABRE, which was found distributed into all the three classes of *DoG*s. ABRE is *G-Box* family motifs with ACGT core, recognized by the bZIP proteins [40]. They function in ABA response through interaction with other ABRE elements or with a coupling element to form an ABA responsive complex (ABRC). The ABRC can mediate ABA dependent gene expression at the promoter region. The enriched presence of ABRE element among the *PeDoGs* confirms their fundamental role in controlling seed dormancy and environmental responsive gene expression. Another *cis*-acting element associated with the phytohormone response found among the *PeDoG* promotors was the GA associated P-box domains. Although P-box domains were not as frequent as the ABRE domains, they form the second most frequent *cis*-element among *PeDoG* genes. P-box domains contain GGTTTT core and are generally found associated with GARE sequences [49]. Among the *PeDoG* genes, not all the genes having P-box motif was found associated with GARE motif, but among the six genes that carried GARE motif had a P-box sequence in its vicinity. This indicated that not all the genes may show GA responsiveness, as observed in the exogenous GA application in the present study. GA is a hormone that regulates physiological activities at different temperatures, and hence *DoG*s associated with GA response are more likely to be influenced by temperature changes. Therefore, GA inducible genes may function in regulating dormancy and stress response as well. As seed dormancy is broken, the *DoGs* facilitate a reduction in ABA concentration, along with a concomitant increase in GA concentration [14, 50], allowing GA to affect the functional expression of *DoGs* in plants. Furthermore, the evidence that exogenous agents such as ABA and GA can induce expression changes in *PeDoG*s points out their regulation of internal stress responses involving these biochemicals. Besides the hormone responsive *cis*-acting elements, there were other elements related to stress response among the *PeDoG* genes. Most common among these were MBS, low temperature-responsive (LTR) element and light response element (Sp1). Among these, MBS was found among 70.8% of the genes indicating it as the most widely available drought inducible element among *PeDoG*s. The next common stress responsive element was the low temperature-responsive LTR motif with 62.5% presence followed by the light responsive Sp1 among 45.8% of the genes. The expression of *PeDoG*s is presumably linked to its gene structure and external environmental factors, such as drought and light, driving an essential impact on the development of the moso bamboo. The expression of *PeDoGs* in different stages of the plant clearly shows that in addition to controlling seed dormancy the *DoG* family also regulates other physiological processes in moso bamboo. For instance, *DoG*s such as *PeDoG15*, *PeDoG20* and *PeDoG22* are seen distributed and strongly expressed in different organs of moso bamboo, indicating their multiple roles. The temporal expression analysis showed that activity of *PeDOG*s decrease during the shoot development due to the dormancy control function mainly acting on the roots, before increasing to high levels of activity in other organs, particularly in the leaves. The temporal expression pattern also showed that the *PeDoG* expression is generally reduced in mature tissues.

The *PeDoG*s expression was not uniform in different plant organs at different stages of growth. Although most genes are constitutively expressed their level varied temporally, indicating differential expression concerning growing conditions. This was evident from the different expression patterns obtained from transcriptome and qRT-PCR samples. In transcriptome data, leaf expression was predominant for several genes; however, a predominance of root expression was seen with qRT-PCR samples. These expression data make it difficult to assign tissue specific gene expression among *PeDoG*s. However, from the combined data, it can be concluded that *PeDoG*s show constitutive expression with a particularly high expression on roots and leaves, two foremost organs that perceive stress signals. Nevertheless, certain genes showed consistent expression patterns such as *PeDoG18*, which showed prominent expression at relatively high levels in the leaves. A similar pattern could be observed with *PeDoG12*, which showed consistently high expression in roots and rhizomes. Among the classes of *PeDoG*s, the class III genes, *PeDoG19* and *PeDoG23*, were found expressed mostly in roots and rhizome, indicating a plausible role of these *DoG* genes in developmental activities of different tissues and organs. Although *DoG*s are implicated in seed dormancy and germination, no data on this is available in moso bamboo so far. Since the growth of moso bamboo is dominated by a prolonged vegetative phase with a flowering cycle of about 60 years, flowering is rare and fruit set is scarce. This is why this study was unable to obtain its seeds for expression analysis and validation of the DoG family in seed development.

## Conclusion

This study provides a bioinformatic analysis of the *PeDoG* genes, validating the mechanisms by which *DoG* family genes regulate seed dormancy. The fact that *PeDoG*s are TFs additionally widens their biological role in moso bamboo beyond a regulator of dormancy. Although their potential role in growth and development and stress response are implicated, any other additional role would be a subject of future investigations. The current information covers their role in ABA regulation, GA response and participation in the SA pathway besides their possible TF functions. These multiple roles of *PeDoG*s suggest that they play a special role in physiological activities of moso bamboo than previously thought of. In future, *PeDoGs* could be subjected to yeast monohybridization to determine their role with bZIP proteins, or yeast two-hybrid crosses to test the relationship between DOG and bZIP and to explore their potential functions in shoots.

## Materials and methods

### Identification and physicochemical characterization of members of the *DoG* gene family of moso bamboo

The general feature format (GFF) sequence file of moso bamboo *(Phyllostachys edulis* (Carr.) Lehaie) genome was downloaded (ftp://parrot.genomics.cn/gigadb/pub/10.5524/100001_101000/100498/) from the Pfam website (http://pfam.xfam.org/). A Hidden Markov Model (HMM) file PF14144.3 from (http://pfam.xfam.org/) was also downloaded from the Pfam database. Using this as a seed model, the local moso bamboo protein database was searched using HMMER3 (http://hmmer.janelia.org/) with E-values set to 1e^−20^. The *PeDoG* gene family members were screened to remove duplicates and obtain candidate gene family members. The gene, CDS, protein sequence, gene structure and chromosome location information of the *DoG* gene family members were further retrieved from the whole genome database in moso bamboo. The information was obtained by ProtParam (https://web.expasy.org/protparam/), WoLF PSORT (https://wolfpsort.hgc.jp/) to analyze the physicochemical properties of each member of the *DoG* gene family online.

### Chromosome distribution and inter- and intra-genomic homologous sequence analysis of the *PeDoG*s

To study the interspecific covariance homology relationships between Moso bamboo, rice and *Arabidopsis*, corresponding *DoG* sequence information were downloaded from the rice database (http://rice.plantbiology.msu.edu) and TAIR (https://www.arabidopsis.org/), respectively; MCScanX was leveraged to obtain the intra- and interspecific covariance relationships of the *DoG* family [51], and the intra- and interspecific covariance results were visualized using the TBtools software, Amazing Super Circos, and Multiple synteny plot, respectively (https://github.com/CJ-Chen/ TBtools).

### Evolutionary analysis of the *PeDoG* family

After selecting the protein sequences of the *DoG* family members of moso bamboo and rice, ClustalW multiple alignments was applied to construct the phylogenetic trees of the *DoG* family genes using the neighbor-joining method in MEGA 7.0 (http://www.megasoftware.net) software with a duplicate test value set to 1000 [52]. The best-fit alternative model (nuclear General "Variable time" matrix) was selected automatically by W-IQ-TREE and the tree was then constructed [52]. Due to the emergence of the bZIP structural domain, we performed MUSCLE multiple sequence alignment based on the known Moso bamboo bZIP family protein sequences reported in the literature as a reference [6], again using the MEGA7.0 software proximity method with a self-test value of 1000 sampling, to construct a phylogenetic tree and clarify the evolutionary relationship of *DoG* with the existing bZIP family.

### Analysis of the *DoG* family promoters in moso bamboo

The upstream sequence of the *DoG* gene was extracted from 2000 bp as the identification site for the cis-regulatory element of the promoter region. The promoter sequence cis-regulatory elements of the moso bamboo *DoG* gene were predicted by the online data analysis software PlantCare (http://bioinformatics.psb.ugent.be/webtools/plantcare/html/). The promoters related to plant hormone response and defence stress response were screened, counted, duplicates were removed, visualized and analyzed using TBtools software, and heat maps were drawn.

### Analysis of conserved structural domains and conserved motifs of the *PeDoGs*

To study the structural domains of the *DoGDoGDoGDoG*

### Homology modelling of the three-dimensional protein sequences from the *PeDoGs*

The PDB database (http://www.rcsb. org/) was used to retrieve the homology templates of the protein sequences about the DOG, besides, using the Swiss Model (https://www.swissmodel. expasy. org/) for homology modelling. The obtained protein tertiary structure models were also evaluated by the online software SAVES (https://saves.mbi.ucla.edu/) to perform the measurements. 

### Prediction and enrichment analysis

The *DoG* family transcription factor prediction and family analysis were performed by PlantTFDB 4.0 (http://planttfdb.cbi.pku.edu.cn/) with a TF prediction by a value of 1E^−5^ to identify the transcription factor. Further, the EggNOG database (evolutionary genealogy of genes: Non-supervised Orthologous Groups (http://eggnogdb.embl.de/#/app/home) was used to predict the number of genes of transcription factors in the family. Using the KEGG database (http://www.genome.jp/ kegg/) [53], *DoG* family pathways were predicted. These pathways were mapped by the online website draw.io (https://app.diagrams.net/). The expression of the family genes at different growth stages was analyzed using a hierarchical clustering approach with a single clustering method using Euclidean distance algorithm. To obtain further information on which functions the gene family, GO enrichment analysis was performed using the software Goatools, with Fisher's exact test set to a significant level *p*-value ≤ 0.05.

### Predictive analysis of protein Interaction networks

After constructing gene sets for the *PeDoG* family, protein interactions were predicted using the online software STRING (https://string-db.org/), and the resulting data were imported into the Cytoscape 3.1.0 (http://www.cytoscape.org/) program to map the PPI network of the DoG family.

### Transcriptome data analysis

To analyze the expression pattern of the *PeDoG* family in four different organs of moso bamboo (leaf, root, whip and inflorescence), the expression abundance (transcripts per million reads, TPM) of *DoG* genes was calculated based on four sets of transcriptomic data (ERR105067, ERR105069, ERR105073, and ERR105075) from the EMBL database (http://wwwdev.ebi.ac.uk/) for moso bamboo leaves, inflorescences, whips and roots.

To mine the response of *DoG* to GA hormone, the transcriptome data of blank control and GA2mM, 0 mM-treated moso bamboo root tissues were obtained from the SRA database in NCBI, with the following accession numbers: SRR6171241, SRR6171242, SRR6171243; SRR5710702, SRR5710701, SRR5710700. The expression abundance TPM values of *DoG* genes were calculated. For statistical purposes, each expression TPM value was logarithmically plotted at a base of 2. TBtools was used to create a heat map of gene expression.

### Quantitative Real-time PCR

RNA templates were extracted from the roots, bamboo whips, young leaves and mature leaves of moso bamboo seedlings. A total of nine genes from three different subfamilies were selected and specific primers were designed by Beacon Designer 7.0 software. To detect the expression of *DoGs* qRT-PCR was carried out. The primer sequences are provided in Table 2. The qPCR mix consisted of 3.7 μL of TB Green II, 0.8 μL of upstream and downstream primers, 0.5 μL of cDNA and ddH2O to make up a total of 10 μL, which was repeated four times, and NTB was used as the reference gene. The PCR reactions were performed on a Takra PCR (TP800) instrument. The procedure was as follows: pre-denaturation at 95 °C for 3 min, denaturation at 95 °C for 10 s, denaturation at 60 °C for 10 s, extension at 72 °C for 20 s, 40 cycles; the lysis curve was measured from 60 °C to 95 °C. The results were analyzed for expression calculations using the 2 ^−∆∆Ct^ method [54].

**Table 2:** Primer’s information used for qPCR of *DoG* family in *P. edulis* (Moso bamboo)

Accession number	Gene number	Sequence (5'-3')	Annealing temperature of PCR (℃)	Amplicon length (bp)
PH02Gene44830.t1	PeDOG6-F	GGAGAAGACAGACAAGAA	76.5	90
	PeDOG6-R	CGAATGAAACCACTCAAC		
PH02Gene37785.t1	PeDOG9-F	AGCTGTCCAACCTTGAAG	76	91
	PeDOG9-R	CCTAAGACTGTCAATCCGT		
PH02Gene37919.t1	PeDOG12-F	CAGACATTGATGAGAGGAA	76.7	117
	PeDOG12-R	AGTATTCTATGAAGCAGCAG		
PH02Gene39868.t1	PeDOG14-F	GGGAATGGAAACGACAAC	89.5	151
	PeDOG14-R	CAAGAAGCTCACGACCTA		
PH02Gene16035.t1	PeDOG18-F	GCACGATGGTTGGAAGAA	75.1	95
	PeDOG18-R	TAGAGGCAACACAACAACT		
PH02Gene31633.t1	PeDOG19-F	TAGGCAACAGGGTGTGTA	80.1	93
	PeDOG19-R	ACAAACTCTACCTCATGCG		
PH02Gene24017.t3	PeDOG21-F	AACAAGGTCCTGAGAAGA	77.9	90
	PeDOG21-R	TAGGTCGTTGATCTCTGT		
PH02Gene47106.t1	PeDOG23-F	CACTACGAGAGGTTGTTC	82.2	108
	PeDOG23-R	GTGTAGAAGGAGACCTAG		
PH02Gene36596.t1	PeDOG24-F	AGAAGGTCCTCAGAAGATT	77.4	90
	PeDOG24-R	TAGGTCGTTGATCTCTGTT		
Nucleotide tract-binding protein	NTB-F	TCTTGTTTGACACCGAAGAGGAG	60	133
	NTB-R	AATAGCTGTCCCTGGAGGAGTTT		

### Time series expression analysis of the *PeDoGs*

STEM analysis with the tool Short Time-series Expression Miner, developed by Ernst and Bar-Joseph [55] (Version 1.3.11) in the NCBI database (https://www.ncbi.nlm.nih.gov/geo/). Twenty-four transcriptome data were downloaded GEO: GSM2810849 and the TPM of the *DoG* family were calculated to analyze the expression patterns of short time sequences at eight time points, observing the changes in gene expression at each separately calculated TPM value of the *DoG* genes at each time point, showing the trend of expression over a period for this gene family. The STEM temporal clustering algorithm was used to group each gene into its most similar trend; the number of temporal patterns was set to 10 and significance was counted by a comparison of the expected number of genes with the actual number of genes in each clump. The significance threshold (p-value) for determining a significant enrichment trend was 0.001 [56].

### Subcellular localization of DOG proteins

To verify the subcellular localization of DOGs protein, the ORF of *PeDOG14* with no terminator codon was reamplified with ORF-F and OR-R primers harboring Xbar1 and SmaI1 sites, respectively. The amplified product was further digested and cloned in the p1300-GFP vector with MAS promotor at the same sites, as a N-terminal fusion protein. Primers used in the amplification of ORF are listed in Table 2. Further, the confirmed recombinant vectors were transformed into *Agrobacterium tumefaciens* strain GV3101. Tobaccos (*Nicotiana benthamiana*) were cultivated in a greenhouse at 24 °C for about one month. The *Agrobacterium* cell with the p1300-PeDOG14-GFP construct) suspension was infiltrated in tobacco leaves and GFP expression was visualized under the confocal microscope (Olympus, Tokyo, Japan) at 480 to 515 nm wavelength after a 72-h incubation period [57].

## Supplementary Information

**Additional file 1: Table S1. **Distribution of the DOG family in the bZIP family. 

**Additional file 2:** **Table S2.** *Cis*-element analysis. 

**Additional file 3:** **Table S3.** KEGG signaling pathway.

**Additional file 4: Table S4. **GO enrichment analysis data. 

**Additional file 5:** **Table S5.** Protein-protein interaction (PPI) data.

## Data Availability

All data generated or analysed during this study are included in this published article and its supplementary information files. The general feature format (GFF) sequence file of moso bamboo (*Phyllostachys edulis*) used in this study are available at ftp://parrot.genomics.cn/gigadb/pub/10.5524/100001_101000/100498/ and in the Pfam website (http://pfam.xfam.org/). The sequences used for the interspecific covariance homology relationships between Moso bamboo, rice and *Arabidopsis*, corresponding DoG sequence information are available in the rice database (http://rice.plantbiology.msu.edu) and TAIR database (https://www.arabidopsis.org/), respectively. The transcriptome data analysed during the current study are available in the EMBL database (http://wwwdev.ebi.ac.uk/) under accession number ERR105067, ERR105069, ERR105073, and ERR105075 and in the NCBI database (https://www.ncbi.nlm.nih.gov/geo/) under GEO accession number GSM2810849.
