# Correction: Genome-wide identification and expression characterization of the *DoG* gene family of moso bamboo (*Phyllostachys edulis*)

**DOI:** 10.1186/s12864-023-09130-w

**Published:** 2023-03-17

**Authors:** Zhang Zhijun, Yu Peiyao, Huang Bing, Ma Ruifang, Kunnummal Kurungara Vinod, Muthusamy Ramakrishnan

**Affiliations:** 1 State Key Laboratory of Subtropical Forest Cultivation, Zhejiang A&F University, Lin’an, Hangzhou, 311300 Zhejiang China; 2 School of Forestry and Biotechnology, Zhejiang A&F University, Lin’an, Hangzhou, 311300 Zhejiang China; 3 Division of Genetics, ICAR - Indian Agricultural Research Institute, New Delhi, 110012 India; 4 Co-Innovation Center for Sustainable Forestry in Southern China, Nanjing Forestry University, Nanjing, 210037 Jiangsu China; 5 Bamboo Research Institute, Nanjing Forestry University, Nanjing, 210037 Jiangsu China

**Correction: BMC Genomics 23, 357 (2022)**

**https://doi.org/10.1186/s12864-022-08551-3**

Following publication of the original article [1], a typesetting error was identified in the **Analysis of conserved structural domains and conserved motifs of the** ***PeDoGs*** section.

The paragraph currently reads:

To study the structural domains of the *DoGDoGDoGDoG*

The paragraph should read:

To study the structural domains of the *DoG* family of moso bamboo, the GFF annotation file of the whole genome of moso bamboo was used to extract the structural information of the moso bamboo DoG family genes. The structural domains of the moso bamboo DoG family were analyzed by NCBI Conserved Domain (https://www.ncbi.nlm.nih.gov/cdd/), and the gene structures and protein structural domains were mapped by TBtools software. The conserved motifs of the DoG family of Moso bamboo were predicted by using the online amino acid conserved motif analysis software MEME (http://meme-suite.org/tools/meme) with the following parameter settings: the motif discovery mode was classical with a predicted number of motifs of 5, and each motif occurred 0 or 1 times.

The original article [1] has been corrected.
